# The rational dose for MaXingShiGan decoction is crucial for its clinical effectiveness in treating bronchial pneumonia: three randomized, double-blind, dose-parallel controlled clinical studies

**DOI:** 10.3389/fphar.2023.1279519

**Published:** 2023-11-23

**Authors:** Xuedong An, Changren Shi, Yaowei Han, Xinmin Li, Lijing Dong, Yan Li, Hui Chen, Yushui Wang, Jinsong Li, Geli Liu, Fengmei Lian, Rong Ma, Xiaolin Tong

**Affiliations:** ^1^ Guang’anmen Hospital of China Academy of Chinese Medical Sciences, Beijing, China; ^2^ The First Affiliated Hospital of Tianjin University of Traditional Chinese Medicine, Tianjin, China; ^3^ Tianjin Hangu District Traditional Chinese Medicine Hospital, Tianjin, China; ^4^ The Second Affiliated Hospital of Tianjin University of Traditional Chinese Medicine, Tianjin, China; ^5^ Tianjin Nankai Hospital, Tianjin, China; ^6^ Tianjin Municipal People’s Hospital, Tianjin, China; ^7^ General Hospital of Tianjin Medical University, Tianjin, China

**Keywords:** MaXingShiGan decoction, bronchial pneumonia, dose-effect, traditional Chinese medicine, clinical study

## Abstract

**Objective:** Evaluate the impact of adjusting the overall dose, Gypsum Fibrosum [Mineral; Gypsum] (ShiGao, SG) dose, and *Prunus armeniaca* L. [Rosaceae; Semen Armeniacae Amarum] (KuXingRen, KXR) dose on the efficacy of MaXingShiGan Decoction (MXSG) in treating children with bronchial pneumonia (Wind-heat Blocking the Lung), in order to provide strategy supported by high-quality evidence for the selection of rational clinical doses of MXSG.

**Methods:** Based on the basic dose of MXSG, we conducted three randomized, double-blind, dose parallel controlled, multicenter clinical trials, involving adjustments to the overall dose, SG dose, and KXR dose, and included 120 children with bronchial pneumonia (Wind-heat Blocking the Lung) respectively. And the patients were divided into low, medium, and high dose groups in a 1:1:1 ratio, with 40 cases in each group. The intervention period lasted for 10 days. The primary outcome was the clinical cured rate, while the secondary outcomes included the effectiveness in alleviating major symptoms of bronchial pneumonia (including fever, cough, dyspnea, and phlegm congestion). And the occurrence of adverse events was recorded.

**Results:** We first recorded and analyzed the baseline characteristics of the three studies, including age, gender, height, and so on. The results indicated that there were no significant differences among the dose groups within each study. For the study adjusting the overall dose of MXSG, the results showed that both the medium-dose group and high-dose group had significantly higher clinical cured rates compared to the low-dose group (Chi-square value 9.01, *p* = 0.0111). However, there was no significant benefit between the high-dose group and the medium-dose group (81.58% vs. 81.08%). Regarding phlegm congestion, excluding fever, cough, and dyspnea, both the medium-dose group and high-dose group had significantly higher clinical cured rates than the low-dose group (Chi-square value 6.31, *p* = 0.0426), and there was no significant benefit between the high-dose group and the medium-dose group (69.23% vs. 75.00%). A total of 5 adverse events were observed, of which only 1 case in the medium-dose group was possibly related to the experimental medication. For the study adjusted the SG dose in MXSG, the results showed that the high-dose group had the highest clinical cured rate, but the inter-group difference was not statistically significant (Chi-square value 3.36, *p* = 0.1864). The area under the curve (AUC) for cough in the medium-dose group was significantly lower than in the low-dose group and high-dose group (F-test value 3.14, *p* = 0.0471). Although no significant differences were observed in fever and dyspnea among the groups, the AUC in the high-dose group was lower than in the medium-dose and low-dose groups. In comparing the complete defervescence time, both the high-dose group (*p* < 0.0001) and the medium-dose group (*p* = 0.0015) achieved faster than the low-dose group. The high-dose group slightly outperformed the medium-dose group (0.50 (0.50, 0.80) vs. 0.80 (0.40, 1.40)), although the difference was not significant. In the medium-dose group, 1 adverse event was observed, but it was not related to the experimental medication. For the study adjusted the KXR dose in MXSG, the results showed that both the medium-dose group and high-dose group had significantly higher cured rates compared to the low-dose group (Chi-square value 47.05, *p* < 0.0001). However, there was no significant benefit comparing the high-dose group to the medium-dose group (90.00% vs. 92.50%). Regarding clinical symptoms, the results indicated that for cough (F-test value 3.16, *p* = 0.0460) and phlegm congestion (F-test value 3.84, *p* = 0.0243), the AUC for both the medium-dose group and high-dose group were significantly lower than in the low-dose group. Although there was benefit in the high-dose group compared to the medium-dose group, it was not statistically significant. No adverse events were observed during the study period.

**Conclusion:** The synthesis of the three conducted clinical studies collectively indicates that for children with bronchial pneumonia (Wind-heat Blocking the Lung), the basic clinical dose of MXSG may represents an optimal intervention dose based on the accumulated clinical experience of doctors. If the dose is insufficient, the clinical effects might be compromised, but using a higher dose does not significantly enhance benefits. Concerning different symptoms, increasing the overall formula’s dose has a favorable impact on improving phlegm congestion, increasing the SG is effective in improving symptoms such as fever, cough, and dyspnea, while higher dose of KXR is effective in alleviating cough and phlegm congestion. These findings suggest that for MXSG, achieving the optimal intervention dose is crucial to achieve better clinical efficacy. For the SG and KXR, if certain symptoms are more severe, increasing the dose can be considered within safe limits, can lead to significant clinical benefits in symptom improvement. This also explains why the dose of MXSG might vary among clinical doctors, while maintaining a balance between safety and effectiveness. Of course, our study is still exploratory clinical trials, and further studies are needed to confirm our findings.

**Clinical Trial Registration:** https://www.chictr.org.cn/index.html; Identifier: ChiCTR-TRC-13003093, ChiCTR-TRC-13003099.

## 1 Introduction

In 2019, the Global Burden of Disease (GBD) study indicated that lower respiratory infections, including pneumonia and bronchitis, affected nearly 500 million people worldwide (Walker et al., 2013; GBD, 2019 Diseases and Injuries Collaborators, 2020). Among them, bronchopneumonia, one of the leading causes of death in children under 5 years old, characterized by acute inflammation of the bronchial mucosa caused by biological or non-biological factors, is a common ailment in children and infants, which characterized by symptoms such as fever, cough, phlegm obstruction, and breathing difficulties (Berlucchi et al., 2014; Chang et al., 2014; Zhang et al., 2018; Ye et al., 2021), which imposes a significant economic burden on families and healthcare systems (Nair et al., 2013). Factors contributing to bronchopneumonia include poor air quality, underdeveloped respiratory systems in children, compromised immune function, and malnutrition (Bradley et al., 2011; Zec et al., 2016; Liu et al., 2020). Among the primary causative agents of bronchopneumonia, *mycoplasma* pneumoniae, bacteria, and viruses play significant roles (Zhang et al., 2007). Treatment strategies for pneumonia mainly involve symptomatic relief, anti-infective therapy, and prevention of complications. Despite the widespread use of infant vaccines that have substantially reduced hospitalization rates due to childhood pneumonia, it remains a major contributor to child mortality (Williams et al., 2002; Black et al., 2010). Notably, glucocorticoids and antibiotics are common therapeutic agents for pneumonia; however, long-term use of these drugs has led to drug resistance and adverse reactions (Ling et al., 2020).

Originating from the “Shang Han Lun,” MaXingShiGan Decoction (MSXG) is a traditional Chinese formula composed mainly of *Ephedra sinica* Stapf [Ephedraceae; HERBA EPHEDRAE] (MaHaung, MH), *Prunus armeniaca* L. [Rosaceae; Semen Armeniacae Amarum] (KuXingRen, KXR), Gypsum Fibrosum [Mineral; Gypsum] (ShiGao, SG), and *Glycyrrhiza glabra* L. [Fabaceae; RADIX GLYCYRRHIZAE] (GanCao, GC). It itself and the derivative formulas such as Lianhua Qingwen Capsules, Han Shi Yi Formula, QingFeiPaiDu Decoction, are commonly employed to treat upper respiratory infections, acute bronchitis, pneumonia, bronchial asthma, and played a pivotal role in the management of the COVID-19 pandemic in China (Zhu and Liu, 2004; Liao et al., 2017; Wang et al., 2020a; Wang et al., 2020b; Tian et al., 2020; Xiao et al., 2020). Additionally, a prior randomized, double-blind, placebo-controlled, multicenter clinical study that we conducted demonstrated the efficacy of MSXG in effectively treating community-acquired childhood pneumonia and significantly improving fever and other clinical symptoms (Zheng et al., 2022).

For traditional Chinese formulas, in addition to the composition of the ingredients, dose is a crucial factor influencing their therapeutic effects, for dose affects the blood concentration of the herbal medicine and can impact the absorption of other components. For instance, KXR and SG can influence the content of ephedrine, a main active component of MH (Liang et al., 2007). GC and KXR can enhance the solubility of SG in water (Guo et al., 2010). Due to variations in individual constitution, metabolism, and illness severity, the question arises: how should the dose of MSXG be adjusted to achieve optimal efficacy while ensuring safety? Should the dose of the entire formula be increased, or is it sufficient to adjust the dose of a single ingredient? Traditional Chinese medicine (TCM) doses often derive from the clinical experience of doctors, which can be subjective and lack high-quality evidence supporting the scientific basis for dose adjustments. This underscores the critical importance of rational clinical application of MSXG.

To address these issues, we conducted three clinical studies. The first directly divided MSXG into low, medium, and high dose groups to observe the varying efficacy among these groups. This was done to evaluate the clinical significance of adjusting the entire formula’s dose. Subsequently, considering the distinct effects of different ingredients in MSXG—such as the use of KXR mainly for treating cough and SG primarily for temperature reduction—we designed two separate clinical studies. These studies aimed to adjust the dose of KXR and SG respectively and assess the impact of adjusting individual ingredients on clinical efficacy.

## 2 Materials and methods

The studies were carried in Guang’anmen Hospital of China Academy of Chinese Medical Sciences, the First Affiliated Hospital of Tianjin University of Traditional Chinese Medicine, the second Affiliated Hospital of Tianjin University of Traditional Chinese Medicine, Tianjin Nankai Hospital, Tianjin Hangu District Traditional Chinese Medicine Hospital, Tianjin People’s Hospital, Tianjin Medical University General Hospital. The studies had obtained approval from the ethics committee of Guanganmen Hospital, Chinese Academy of Chinese Medical Sciences (Ethics approval number: 2010–35), and has been registered on Chinese Clinical Trial Registry (https://www.chictr.org.cn/index.html, ChiCTR-TRC-13003093, ChiCTR-TRC-13003099).

### 2.1 Quality control and safety experiments of MSXG

We have established the detailed decoction process for the MSXG, which involves taking traditional Chinese herbs, adding 8 times their weight in water, soaking for 30 min, bringing to a boil, and then simmering on low heat for 40 min. Afterward, it is allowed to cool, filtered through gauze, and the resulting liquid is collected. Initially, we determined the content of the purchased herbs according to the standards set by the Chinese Pharmacopoeia to decide which herbal materials to use in the clinical study.

We employed both HPLC (High-Performance Liquid Chromatography) and titration methods to determine the content of MSXG. The results showed that the similarity of fingerprint profiles for 10 batches of samples was all greater than 0.95, indicating the stability of the preparation process for MSXG. Specific fingerprint peaks were identified, including ephedrine, pseudoephedrine, amygdalin, glycyrrhizin, and glycyrrhetinic acid. Our data on the fingerprint spectrum of Mahuang Xingren Gan Tang has been published. (*Wei Huizhen, Wang Xin, Wang Yuesheng, et al. Study on Multi-Wavelength Switching Fingerprint Spectrum of Mahuang Xingren Gan Tang. Journal of Traditional Chinese Medicine and Traditional Chinese Medicine Materials, 2012, 23(01): 60–62.*)

Before conducting clinical research, we conducted two animal experiments. The first experiment detected the components absorbed into the bloodstream, including ephedrine or pseudoephedrine, methyl ephedrine, glycyrrhizin, glycyrrhetinic acid, and isoglycyrrhizin. The second experiment evaluated the toxicity of MSXG, and the results showed that there were no significant toxic reactions observed at a maximum single oral dose equivalent to 87.5 human daily doses (Supplementary Appendix).

### 2.2 Study design

All 3 clinical studies conducted randomized, double-blind, dose-controlled, multi-center design. Regarding the sample size of the studies, we employed adaptive design. Each study aimed to recruit a total of 120 eligible patients according to the inclusion criteria, with 40 cases in each group. Depending on the statistical results, the number of cases could be increased by 30–60 cases as deemed appropriate.

In study 1, a total of 120 patients were enrolled and randomly divided into three different dose groups for MSXG: low, medium, and high, with 40 patients in each group.

In study 2, a total of 120 patients were enrolled, and only the dose of SG in MSXG was adjusted. They were divided into three different dose groups: low, medium, and high, with 40 patients in each group.

In Study 3, a total of 120 patients were enrolled, and only the dose of KXR in MSXG was adjusted. They were divided into three different dose groups: low, medium, and high, with 40 patients in each group.

### 2.3 Patients

#### 2.3.1 Inclusion criteria

The patient meets the diagnostic criteria for bronchial pneumonia (Wind-heat Blocking the Lung) in the “Prevention and Treatment of Children’s Four Diseases, Prevention and Treatment of Pediatric Pneumonia” and “Zhu Futang Practical Pediatrics” (seventh edition). The diagnostic criteria for TCM syndromes were referenced from “Diagnostic Criteria for Traditional Chinese Medicine Diseases and Syndromes and Efficacy Standards: Pediatric Diseases” (Supplementary Appendix).

1) The course of pneumonia should not exceed 48 h.
2) Within 24 h before enrollment maximum body temperature ≥38.5°C.
3) Age 3–6 years old.
4) Weight ≥14 kg.
5) White blood cell count ≤10×10^9^/L, neutrophil ratio less than 70%.
6) Normal C-reactive protein.
7) Hospitalized patients.
8) Legal guardian of the child Informed and signed informed consent.

#### 2.3.2 Exclusion criteria

1) Patients with heart failure, respiratory failure, toxic encephalopathy, exudative pleurisy and other comorbidities.
2) Clearly complicated bacterial infection.
3) Complicated with serious primary diseases such as heart, liver, kidney and hematopoietic system, mentally ill patients, if clinically significant arrhythmia, alanine aminotransferase more than double the upper limit of normal, serum creatinine >150 μmol/L, urea >10 mmol/L, or/and proteinuria >+, or/and erythrocyte urine >+.
4) According to the judgment of the investigator, there are other diseases that reduce the possibility of enrolling or complicate enrollment.
5) Those who could not cooperate or are participating in clinical trials of other drugs.
6) Allergic constitution (allergic to more than two types of substances) or known those who were allergic to the components of this preparation.
7) According to the judgment of the doctor, those who were likely to be lost to follow-up.

### 2.4 Interventions

All patients, in addition to receiving azithromycin (Zithromax, Pfizer Pharmaceuticals, 0.5 mg per dose, 5–10 mg/kg·d, once daily) as the foundational treatment, were administered different doses of MXSG based on their respective assigned groups. (Supplementary Tables SA1–SA3).

The MXSG was composed of 4 herbs: *Ephedra sinica* Stapf (MaHaung, MH), *Prunus armeniaca* L. (KuXingRen, KXR), Gypsum fibrosum (ShiGao, SG), and *Glycyrrhiza uralensis* Fisch. (GanCao, GC). And the basic dose of MXSG was MH 6g, KXR 9g, SG 24g, GC 6g, which were supplied by Yanjing Herb Pharmaceutical Co., Ltd., (Beijing, China), decocted and distributed uniformly by the First Affiliated Hospital of Tianjin University of Traditional Chinese Medicine, and conform to the Chinese Pharmacopoeia (2010 edition).

For Study 1, the doses for each group were as follows:

- Low-dose group: MH 3g, KXR 3g, SG 12g, GC 3g.
- Medium-dose group: MH 6g, KXR 6g, SG 24g, GC 6g.
- High-dose group: MH 9g, KXR 9g, SG 36g, GC 9g.

For Study 2, the doses for each group were:

- low-dose group: MH 6g, KXR 6g, SG 16g, GC 6g.
- Medium-dose group: MH 6g, KXR 6g, SG 24g, GC 6g.
- high-dose group: MH 6g, KXR 6g, SG 36g, GC 6g.

For Study 3, the doses for each group were:

- low-dose group: MH 6g, KXR 3g, SG 16g, GC 6g.
- Medium-dose group: MH 6g, KXR 6g, SG 24g, GC 6g.
- high-dose group: MH 6g, KXR 9g, SG 24g, GC 6g.

Uniform labelling format for the study drugs, including contents: name of clinical trial drug (for clinical research only), medication method, specification, storage conditions, drug number, expiry date, drug supply unit, matters needing attention. The observing physician should distribute the drugs according to the order of visit and the drug number of each patient. The drug number should not be selected, and the drug number would remain unchanged throughout the trial. Each patient would be provided with enough study drugs of the same drug number for 10 days. Strict management and use of test drugs and control drugs, each participating unit to establish a strict test department of specially assigned custody, distribution system. The First Affiliated Hospital of Tianjin University of Traditional Chinese Medicine delivered each group of TCM decoction directly to the special custodian of each hospital department, and establish a perfect drug reception procedure. The drug should not be heated or exposed to direct sunlight during delivery, and freezing is prohibited. Unused test drugs should be stored in the refrigerator at 2 ∼ 8°C. The study drugs are the responsibility of the investigator, and the investigator should not transfer the investigational drugs to any non-clinical trial participants. The investigator must ensure that the drug is used only in the subjects of the clinical trial, that the dose and administration are in accordance with the trial protocol, and that any remaining drug is withdrawn.

## 3 Outcomes

### 3.1 Baseline characteristics

The gender, age, height, weight, medical history, symptoms of the patients were recorded.

### 3.2 Effect outcomes

#### 3.2.1 Primary outcome

The clinical cured rate (number of clinical cured patients/total number of patients * 100%) as the primary outcome, the evaluation criteria of disease efficacy as follows:

1) Clinical cured: ①the fine moist rales disappeared in the lung auscultation; ②the fever was completely relieved; ③ the dyspnea disappeared.
2) Not cured: Those who did not reach the clinical cured standard.
3) Invalid: ① the daily maximum body temperature dropped by less than 0.5°C; ② the symptoms and signs have no obvious change or aggravation. Both conditions are met at the same time.

#### 3.2.2 Second outcomes

We first evaluate the main clinical symptoms of bronchitis, including fever, cough, dyspnea, and phlegm congestion.

For study 1, we used the symptom disappearance rate, and the evaluation criteria were: Clinical cured: Symptoms disappear after treatment. Significant effect: After treatment, the severity of symptoms decreased by 2 levels, from severe to mild. Effective: After treatment, the severity of symptoms decreases by 1 level, from severe to moderate, or from moderate to mild. Invalid: There is no change before and after treatment. (Supplementary Table SA4).

For Study 2 and Study 3, we considered that adjusting for a single drug may result in relatively small differences in efficacy between groups. Therefore, the improvement in symptom (fever, cough, phlegm obstruction, dyspnea) was evaluated with the area under the curve (AUC) between the symptom and time.

We observed during study that some patients had resolved their fever symptoms before the study’s completion. Aimed to comprehensively assess the efficacy of SG in Study 2 regarding fever management, we evaluated the complete defervescence time among the groups. This refers to the time required for the body temperature to return to normal (≤37.2°C) and remain so for more than 24 h after the administration of the drug.

### 3.3 Safety outcomes

Safety outcomes included detection of chest X-ray, white blood cell count and classification, C-reactive protein, *mycoplasma* pneumoniae antibody, and routine stool, urine, electrocardiogram, liver and kidney function.

1) Vital signs: including body temperature, resting heart rate, resting breathing, blood pressure.
2) Laboratory tests: including blood routine (erythrocytes, hemoglobin, leukocytes, neutrophils, lymphocytes, platelets), urine routine (erythrocytes, leukocytes, urine protein, urine sugar), stool routine (leukocytes, erythrocytes), liver and kidney function, including alanine aminotransferase (ALT), aspartate aminotransferase (AST), blood urea nitrogen (BUN), Serum creatinine (Cr).
3) Electrocardiogram.
4) Chest radiograph.
5) Adverse event.

### 3.4 Statistical analysis

All analyses were performed using SAS 9.2. All statistical tests adopt two-sided test. If the *p*-value was less than or equal to 0.05, it would be considered that the tested difference was statistically significant (unless otherwise specified). The description of quantitative indicators would calculate the number of cases, missing number, mean, standard deviation, minimum value, maximum value, median and interquartile spacing. F test/Wilcoxon rank sum test was used to compare the quantitative indexes between groups; For qualitative indexes, chi square test/Fisher exact test was used for comparison between groups.

Full analysis set (FAS): All subjects who had been randomized into groups, taken the test drug at least once, and have post-dose evaluation data constitute the FAS of this trial. Missing data in the efficacy-related portion of the FAS would be supplemented using the last previous observation carried forward (LOCF). FAS was used for the analysis of primary and secondary efficacy measures and was the main dataset for efficacy evaluation in this trial.

Per-protocol analysis (PPS): Subjects who met the inclusion criteria specified by the trial protocol; completed the 6-day planned visit; no drugs or treatments were used during the trial that might affect the evaluation of efficacy; adherence was good (80%–120%).

Safety Set (SS): All subjects who have been randomized into different groups, received the investigational drug at least once, and possess safety evaluation data after drug administration constitute the SS of this trial.

## 4 Results

### 4.1 Baseline characteristics

3 studies each enrolled 120 patients with bronchial pneumonia (Wind-heat Blocking the Lung). For Study 1, a total of 112 patients completed the study, with 39 in the low-dose group, 37 in the medium-dose group, and 36 in the high-dose group. For Study 2, a total of 110 patients completed the study, including 35 in the low-dose group, 37 in the medium-dose group, and 38 in the high-dose group. For Study 3, a total of 100 patients completed the study, with 26 in the low-dose group, 37 in the medium-dose group, and 37 in the high-dose group. The baseline characteristics of the subjects were presented in the Supplementary Tables SA5–SA7, and there were no differences between the groups in each study, including age, gender, height, and so on. Flowcharts for each study are provided in the Supplementary Figures SA1–SA3.

### 4.2 Primary outcome

We first analyzed the effects of MXSG in the treatment of bronchial pneumonia in 3 studies. The results showed that for the FAS, the cured rate was 75.71% (268/354), and for the PPS, the cured rate was 83.13% (266/320). For Study 1, the FAS results revealed that among the patients in the low-dose group, 22 individuals (55.00%) achieved clinical cured, while in the medium-dose group, 31 patients (81.58%) achieved clinical cured, and in the high-dose group, 30 patients (81.08%) achieved clinical cured. There was a significant difference in the comparison between the groups (Chi-square value: 9.01, *p* = 0.0111), indicating that both the medium-dose and high-dose groups had significantly higher clinical cured rates than the low-dose group. The PPS results showed that among the patients in the low-dose group, 22 individuals (56.41%) achieved clinical cured, while in the medium-dose group, 31 patients (86.11%) achieved clinical cured, and in the high-dose group, 30 patients (85.71%) achieved clinical cured. There was a significant difference in the comparison between the groups (Chi-square value: 11.83, *p* = 0.0027), indicating that both the medium-dose and high-dose groups had significantly higher clinical cured rates than the low-dose group.

For Study 2, the FAS results showed that among the patients in the low-dose group, 30 individuals (75.00%) achieved clinical cured, in the medium-dose group, 34 patients (85.00%) achieved clinical cured, and in the high-dose group, 36 patients (90.00%) achieved clinical cured. There was no significant difference in the comparison between the groups (Chi-square value: 3.36, *p* = 0.1864), indicating that there were no significant differences in clinical cured rates among the groups. The PPS results showed that among the patients in the low-dose group, 30 individuals (85.71%) achieved clinical cured, in the medium-dose group, 34 patients (91.89%) achieved clinical cured, and in the high-dose group, 36 patients (94.74%) achieved clinical cured. There was no significant difference in the comparison between the groups (two-sided exact probability, *p* = 0.3760), indicating that there were no significant differences in clinical cured rates among the groups.

**FIGURE 1:**
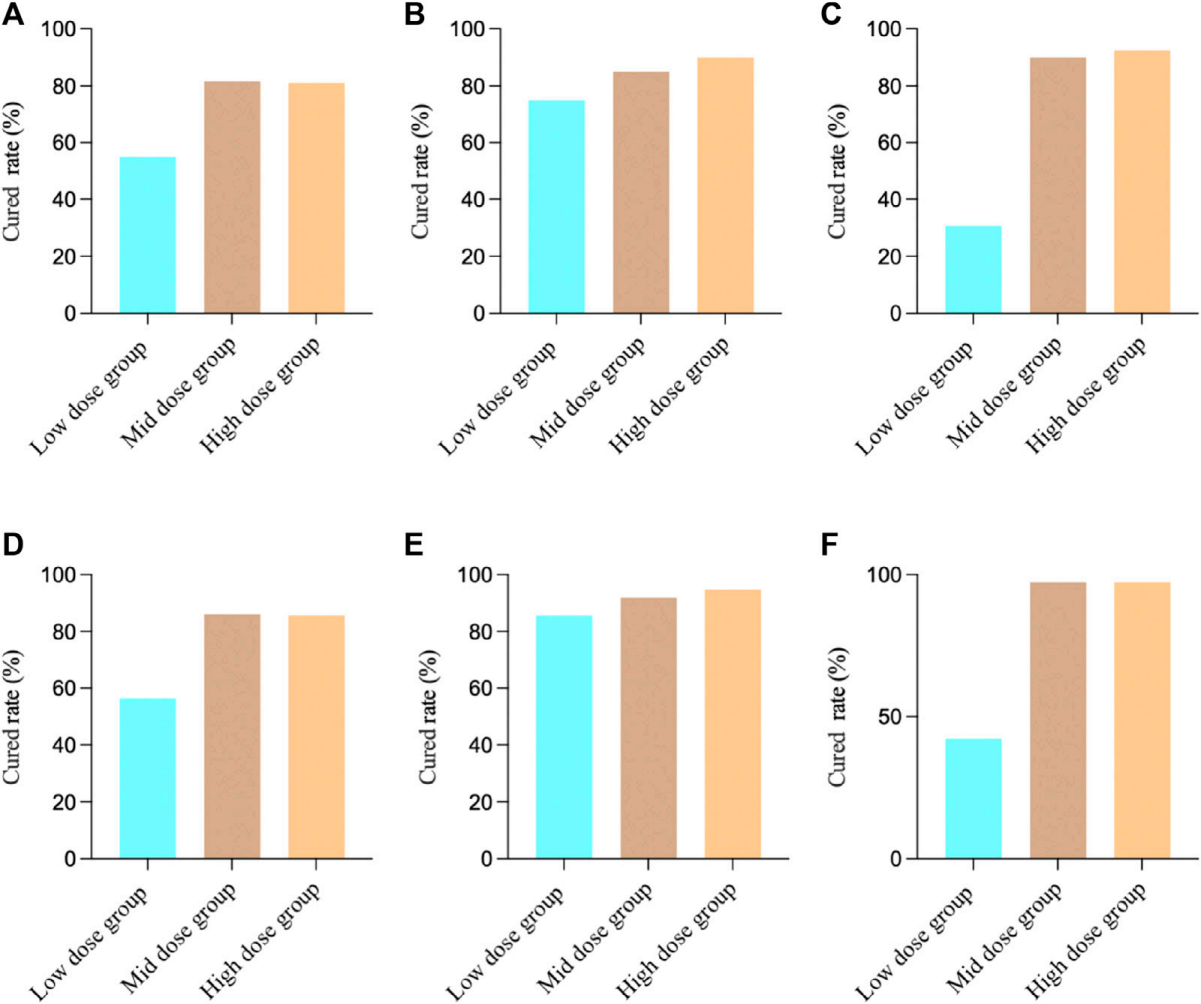
Clinical cured results in different studies. **(A)** Study 1 FAS Results; **(B)** Study 2 FAS Results; **(C)** Study 3 FAS Results; **(D)** Study 1 PPS Results; **(E)** Study 2 PPS Results; **(F)** Study 3 PPS Results.

For Study 3, the FAS results showed that among the patients in the low-dose group, 12 individuals (30.77%) achieved clinical cured, in the medium-dose group, 36 patients (90.00%) achieved clinical cured, and in the high-dose group, 32 patients (92.50%) achieved clinical cured. There was a significant difference in the comparison between the groups (Chi-square value: 47.05, *p* < 0.0001), indicating that both the medium-dose and high-dose groups had significantly higher clinical cured rates than the low-dose group. The PPS results showed that among the patients in the low-dose group, 11 individuals (42.31%) achieved clinical cured, in the medium-dose group, 36 patients (97.30%) achieved clinical cured, and in the high-dose group, 36 patients (97.30%) achieved clinical cured. There was a significant difference in the comparison between the groups (two-sided exact probability, *p* < 0.0001), indicating that both the medium-dose and high-dose groups had significantly higher clinical cured rates than the low-dose group (Figure 1).

### 4.3 Second outcomes

For the FAS in Study 1, the fever results showed that, 36 patients (100.00%) in the low-dose group achieved clinical cured, 33 patients (94.29%) in the medium-dose group, and 34 patients (97.14%) in the high-dose group. There was no significant difference between the groups (two-sided exact probability, *p* = 0.3208). For cough, 19 patients (48.72%) in the low-dose group achieved clinical cured, 28 patients (71.79%) in the medium-dose group, and 24 patients (64.86%) in the high-dose group. There was no significant difference between the groups (Chi-square value: 4.62, *p* = 0.0992). For dyspnea, 14 patients (77.78%) in the low-dose group achieved clinical cured, 10 patients (62.50%) in the medium-dose group, and 18 patients (75.00%) in the high-dose group. There was no significant difference between the groups (two-sided exact probability, *p* = 0.5713). For phlegm obstruction, 19 patients (48.72%) in the low-dose group achieved clinical cured, 27 patients (69.23%) in the medium-dose group, and 27 patients (75.00%) in the high-dose group. There was a significant difference between the groups (Chi-square value: 6.31, *p* = 0.0426).

**FIGURE 2:**
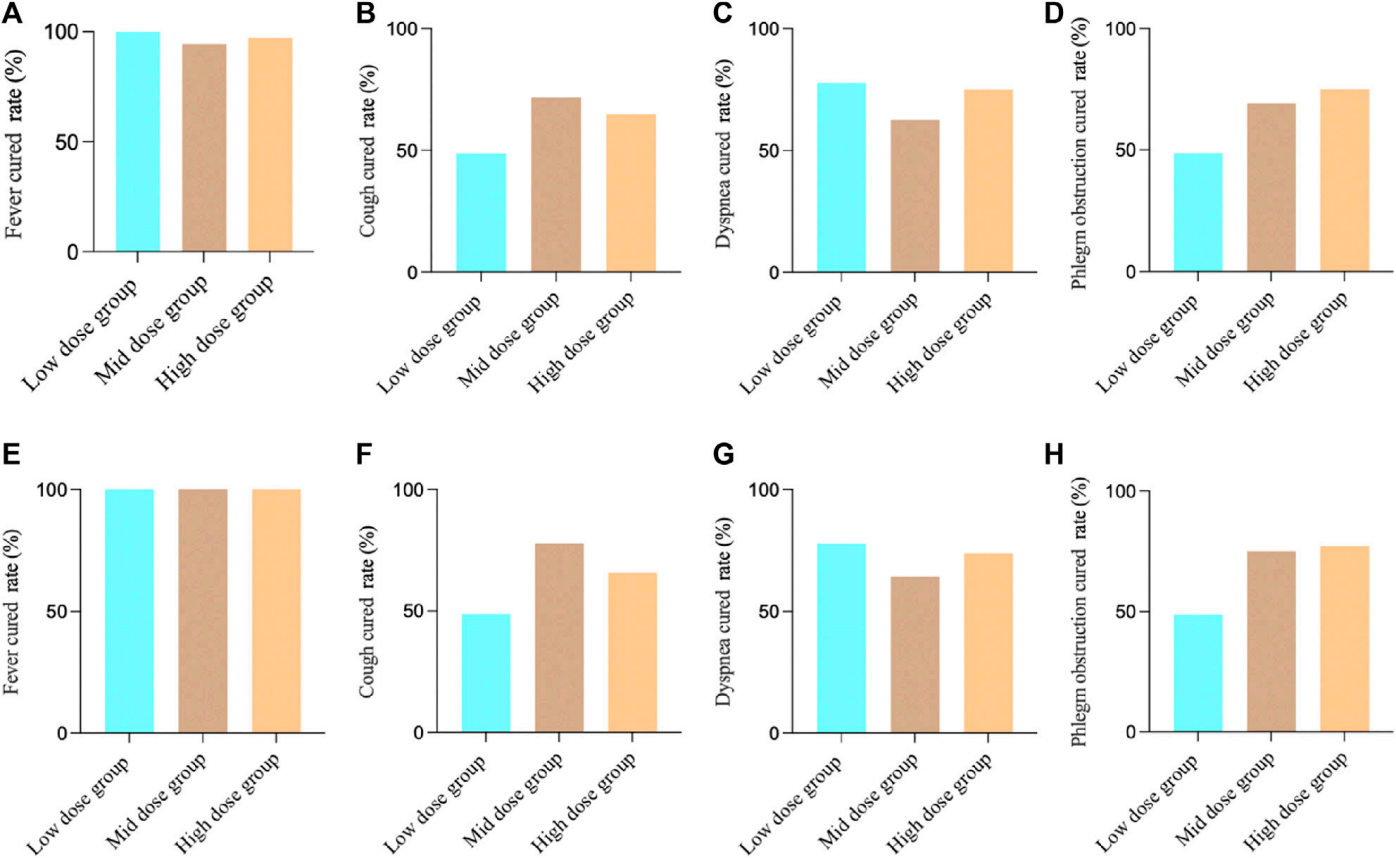
Study 1 results of different symptoms. **(A)** FAS results of fever; **(B)** FAS results of cough; **(C)** FAS results of dyspnea; **(D)** FAS results of phlegm obstruction; **(E)** PPS results of fever; **(F)** PPS results of cough; **(G)** PPS results of dyspnea; **(H)** PPS results of phlegm obstruction.

For the PPS in Study 1, the fever results showed, 36 patients (100.00%) in the low-dose group achieved clinical cured, 32 patients (100.00%) in the medium-dose group, and 33 patients (100.00%) in the high-dose group. There was no significant difference between the groups (*p* = 1.000). For cough, 19 patients (48.72%) in the low-dose group achieved clinical cured, 28 patients (77.78%) in the medium-dose group, and 23 patients (65.71%) in the high-dose group. There was a significant difference between the groups (Chi-square value: 6.93, *p* = 0.0313). For dyspnea, 14 patients (77.78%) in the low-dose group achieved clinical cured, 9 patients (64.29%) in the medium-dose group, and 17 patients (73.91%) in the high-dose group. There was no significant difference between the groups (two-sided exact probability, *p* = 0.6900). For phlegm obstruction, 19 patients (48.72%) in the low-dose group achieved clinical cured, 27 patients (75.00%) in the medium-dose group, and 27 patients (77.14%) in the high-dose group. There was a significant difference between the groups (Chi-square value: 8.46, *p* = 0.0145). (Figure 2).

For the FAS in Study 2, the fever AUC results showed, the low-dose group was 7.60 ± 5.09, the medium-dose group was 7.78 ± 5.72, and the high-dose group was 5.93 ± 4.16. There was no significant difference between the groups (F-test value: 1.65, *p* = 0.1970). For cough, the low-dose group was 20.73 ± 7.20, the medium-dose group was 18.25 ± 4.93, and the high-dose group was 21.53 ± 5.95. There was a significant difference between the groups (F-test value: 3.14, *p* = 0.0471). For dyspnea, the low-dose group was 3.15 ± 4.19, the medium-dose group was 2.98 ± 3.42, and the high-dose group was 1.58 ± 2.55. There was no significant difference between the groups (F-test value: 2.50, *p* = 0.0863). For phlegm obstruction, the low-dose group was 19.70 ± 8.04, the medium-dose group was 17.85 ± 6.65, and the high-dose group was 19.88 ± 6.04. There was no significant difference between the groups (F-test value: 1.04, *p* = 0.3567).

For the PPS in Study 2, the fever AUC results showed, the low-dose group was 7.86 ± 5.35, the medium-dose group was 7.43 ± 5.44, and the high-dose group was 5.39 ± 3.33. There was no significant difference between the groups (F-test value: 2.81, *p* = 0.0649). For cough, the low-dose group was 21.74 ± 6.55, the medium-dose group was 18.86 ± 4.56, and the high-dose group was 21.87 ± 5.74. There was a significant difference between the groups (F-test value: 3.32, *p* = 0.0398). For dyspnea, the low-dose group was 3.29 ± 4.35, the medium-dose group was 3.16 ± 3.48, and the high-dose group was 1.66 ± 2.59. There was no significant difference between the groups (F-test value: 2.47, *p* = 0.0898). For phlegm obstruction, the low-dose group was 20.86 ± 7.71, the medium-dose group was 18.46 ± 6.53, and the high-dose group was 20.18 ± 5.88. There was no significant difference between the groups (F-test value: 1.23, *p* = 0.2962).

**FIGURE 3:**
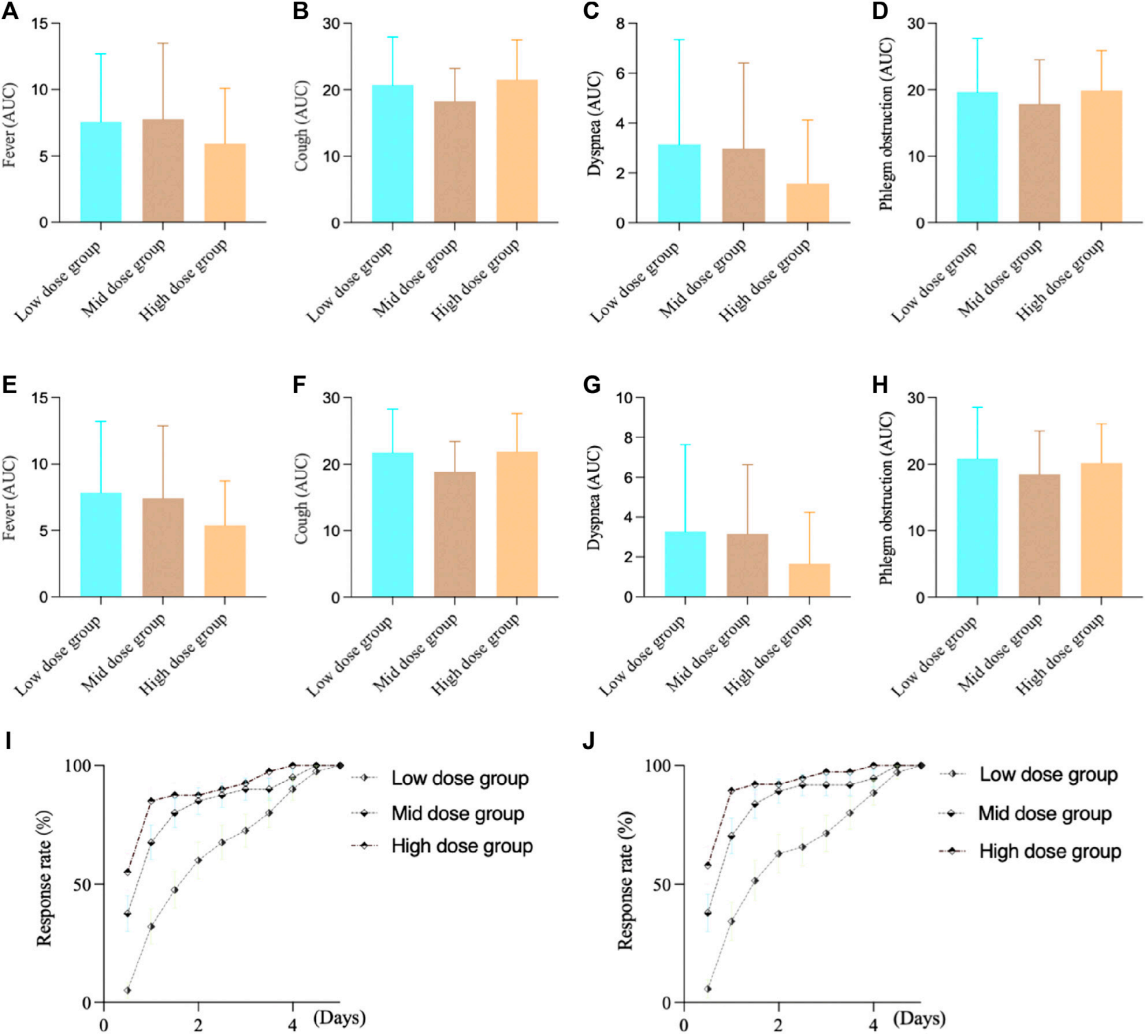
Study 2 results of different symptoms. **(A)** FAS results of fever; **(B)** FAS results of cough; **(C)** FAS results of dyspnea; **(D)** FAS results of phlegm obstruction; **(E)** PPS results of fever; **(F)** PPS results of cough; **(G)** PPS results of dyspnea; **(H)** PPS results of phlegm obstruction; **(I)** FAS results of complete antipyretic time; **(J)** PPS results of complete antipyretic time.

During the study, we observed that many patients experienced complete relief from fever symptoms around 3 days of intervention. Therefore, we compared the time of complete fever resolution among different groups to assess the onset of action of the intervention medication. For FAS, the complete antipyretic time in high dose group (Log-Rank test, statistic 25.38, *p* < 0.0001) and medium-dose group (Log-Rank test, statistic 10.07, *p* = 0.0015) showed statistical differences compared to low-dose group after treatment, and there was no statistical difference between the high and middle dose groups (Log-Rank test, statistic 2.48, *p* = 0.1152). For PPS, the complete antipyretic time in high dose group (Log-Rank test, statistic 30.13, *p* < 0.0001) and medium-dose group (Log-Rank test, statistic 11.17, *p* = 0.0008) showed statistical differences compared to low-dose group after treatment, and there was no statistical difference between the high and middle dose groups (Log-Rank test, statistic 3.34, *p* = 0.07). (Figure 3).

For the FAS in Study 3, the fever AUC results showed, the low-dose group was 1.62 ± 2.65, the medium-dose group was 1.60 ± 2.73, and the high-dose group was 2.10 ± 3.80. There were no significant differences between the groups (F-test value: 0.33, *p* = 0.7171). For cough, the low-dose group was 23.87 ± 8.02, the medium-dose group was 20.75 ± 7.17, and the high-dose group was 20.25 ± 5.33. There was a significant difference among the groups (F-test value: 3.16, *p* = 0.0460). For dyspnea, the low-dose group was 0.08 ± 0.48, the medium-dose group was 0.68 ± 2.25, and the high-dose group was 0.73 ± 2.76. There were no significant differences between the groups (F-test value: 1.18, *p* = 0.3115). For phlegm obstruction, the low-dose group was 23.08 ± 8.64, the medium-dose group was 19.73 ± 7.64, and the high-dose group was 18.68 ± 5.47. There were no significant differences between the groups (F-test value: 3.84, *p* = 0.0243).

**FIGURE 4:**
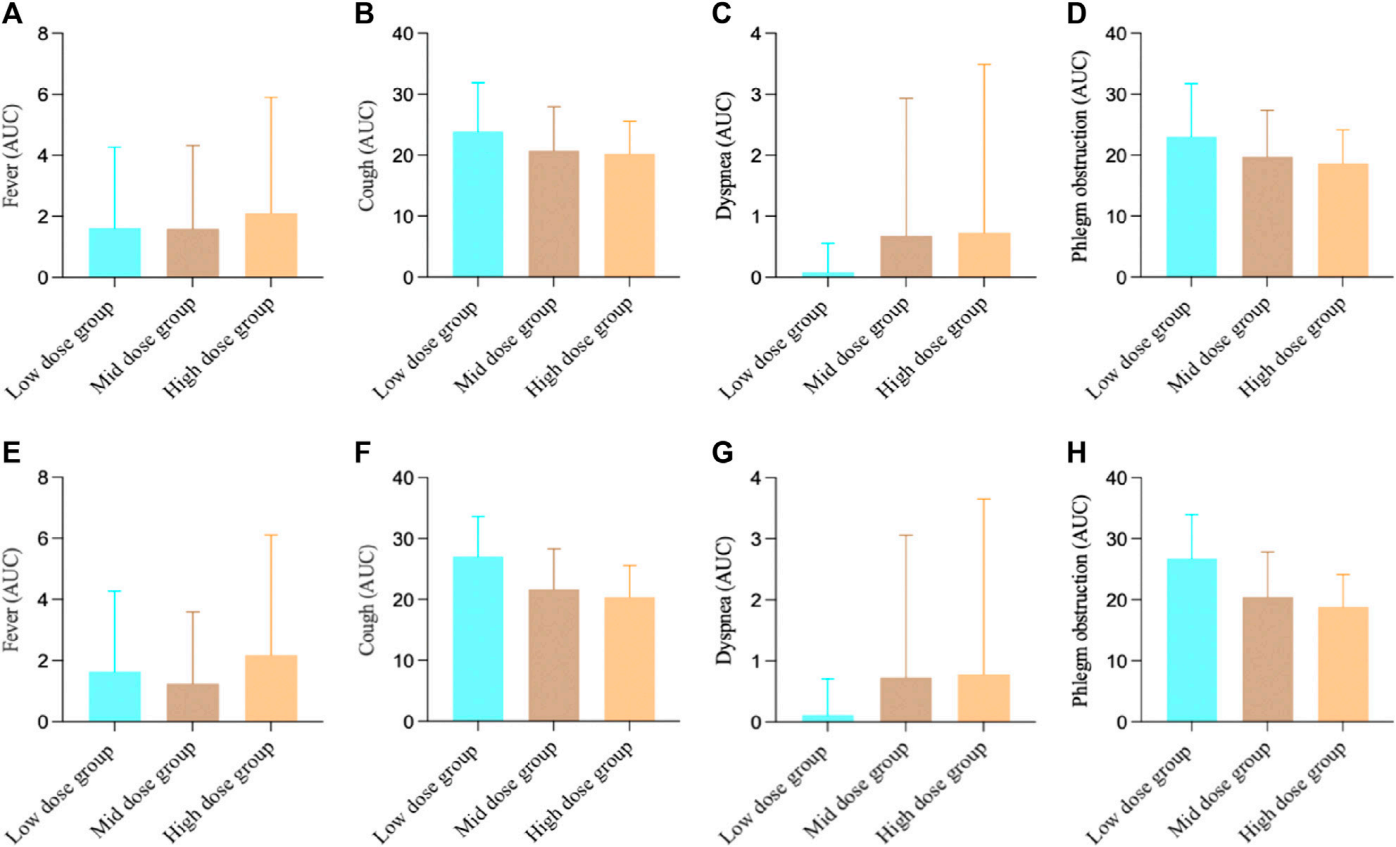
Study 3 results of different symptoms **(A)** FAS results of fever; **(B)** FAS results of cough; **(C)** FAS results of dyspnea; **(D)** FAS results of phlegm obstruction; **(E)** PPS results of fever; **(F)** PPS results of cough; **(G)** PPS results of dyspnea; **(H)** PPS results of phlegm obstruction.

For the PPS in Study 3, the fever AUC results showed, the low-dose group was 1.65 ± 2.62, the medium-dose group was 1.24 ± 2.36, and the high-dose group was 2.19 ± 3.92. There were no significant differences between the groups (F-test value: 0.87, *p* = 0.4219). For cough, the low-dose group was 27.04 ± 6.61, the medium-dose group was 21.68 ± 6.59, and the high-dose group was 20.38 ± 5.16. There was a significant difference among the groups (F-test value: 9.74, *p* = 0.0001). For dyspnea, the low-dose group was 0.12 ± 0.59, the medium-dose group was 0.73 ± 2.33, and the high-dose group was 0.78 ± 2.87. There were no significant differences between the groups (F-test value: 0.77, *p* = 0.4646). For phlegm obstruction, the low-dose group was 26.77 ± 7.18, the medium-dose group was 20.46 ± 7.39, and the high-dose group was 18.81 ± 5.29. There was a significant difference among the groups (F-test value: 11.71, *p* < 0.001). (Figure 4).

### 4.4 Adverse events

In Study 1, a total of 5 adverse events were reported. Among them, there were 2 adverse events in the medium-dose group, with 1 of them possibly related to the investigational drug. In the high-dose group, there were 3 adverse events, but none of them were likely related to the investigational drug. In Study 2, there was a total of 1 adverse event observed, occurring in the high-dose group. However, this adverse event was not likely related to the investigational drug. In Study 3, no adverse events were observed. (Supplementary Table SA8).

## 5 Discussion

Safety and efficacy are two fundamental attributes of therapeutic drugs, including TCMs. As the understanding of treatment outcomes and adverse reactions in TCMs deepens, the dose-response relationship—selecting the appropriate dosage to maximize therapeutic effectiveness while minimizing adverse reactions—is becoming a crucial concern in the modernization of TCM. Consequently, a comprehensive understanding of the accuracy of herbal dose selection is of paramount importance in the daily practice of TCM, ensuring optimal treatment for patients with various ailments (Zha et al., 2015). In clinical practice, for different TCMs and formulations composed of these, clinical applications in TCM have accumulated a considerable amount of experience. However, much of this experience is based on individual expertise and has yet to be validated through high-quality evidence. This is precisely one of the significant aims of conducting this study—to establish a basis for this through rigorous evidence-based research.

In summary, we conducted 3 clinical studies focusing on the commonly used clinical formula MXSG in treating bronchial pneumonia. First, in terms of clinical efficacy, the clinical cured rate of the low-dose group was the lowest among the 3studies. Although there was a slight increase in the clinical cured rate in the high-dose group compared to the medium-dose group, it was not statistically significant. Taking into consideration the clinical components and safety concerns, the medium dose appears to be the optimal dosage for clinical treatment of bronchitis. However, when assessing individual symptoms, in Study 1, the clinical cured rates for cough and phlegm obstruction were higher in both the medium and high-dose groups compared to the low-dose group. Meanwhile, there was no significant clinical benefit observed in the high-dose group compared to the low-dose group. This emphasizes the importance of clinical effectiveness, and increasing the overall dosage might not result in significant clinical benefits. For Study 2 and Study 3, the results indicated that the clinical efficacy of the medium and high-dose groups was significantly better than that of the low-dose group. However, while the high-dose group exhibited some improvement compared to the medium-dose group, the difference was not statistically significant. In terms of clinical symptoms, the AUC results for fever and dyspnea indicated that the high-dose group had the most significant therapeutic effect. When combined with the results of the complete defervescence rate, patients in the high-dose group were more likely to have fever symptoms subside in a shorter time frame. Additionally, no significant drug-related adverse events were observed, suggesting that the rational increase of SG on top of the commonly used dosage can lead to more significant fever-reducing effects. This includes clinical recovery from fever as well as a shorter duration until the disappearance of fever symptoms. In Study 3, the results were consistent with Study 1 and Study 2. Both the medium and high-dose groups had significantly higher clinical cured rates compared to the low-dose group. However, there was no significant clinical benefit observed in the high-dose group compared to the medium-dose group. In terms of clinical symptoms, the results showed that the high-dose group had the most significant therapeutic effect in terms of cough and phlegm obstruction. Both the high and medium-dose groups were superior to the low-dose group. In conclusion, we conducted 3 clinical studies centered around MXSG and primarily investigated how to adjust its dosage during clinical applications. Our findings suggest that the commonly used clinical dosage is the optimal one for the entire formula. If the dosage is not enough, the desired clinical efficacy may not be achieved, and increasing the dosage further may not result in significant clinical benefits. For different herbs within the formula, adjusting the dosage of SG, a cooling agent, does not significantly improve the overall efficacy but does show notable changes in fever symptoms. As for KXR, its adjustment produces more significant improvements in cough symptoms.

As mentioned above, MXSG is used to treat respiratory tract infections, acute bronchitis, pneumonia, bronchial asthma and other lung diseases, as well as H1N1, Corona Virus Disease 2019 (COVID-19) and other pulmonary infectious diseases (Wang et al., 2011), with mechanism of stimulation of beta 2-adrenergic receptors on bronchial smooth muscle, inhibition of neutrophil entry into the airways, and reduction of airway inflammation (Kao et al., 2001), and exerts broad-spectrum antiviral effects by inhibiting viral RNA and protein synthesis (Hsieh et al., 2012). The pharmacodynamic mechanism mainly involves antiviral, alleviating lung inflammation and reducing lung cell apoptosis. *In vivo* animal experiments have shown that MXSG could reduce lung inflammation induced by lipopolysaccharide in a rat model of pneumonia, possibly by regulating the coagulation system (Yang et al., 2020). MXSG colud also inhibit the activation of the high mobility group protein 1/Toll-like receptor 4/nuclear factor kappa-B (NF-κB) signaling pathway and reduce the levels of inflammatory cytokines, thereby alleviating inflammatory damage (Fei et al., 2019). Clinical studies have confirmed that MXSG in combination with other drugs could significantly improve the antipyretic effect, such as Oseltamivir and MXSG + Yinqiaosan alone or in combination could shorten the time of fever in patients with H1N1 influenza virus infection. These data suggest that MXSG + Yinqiaosan could be used as an alternative treatment for H1N1 influenza virus infection (Wang et al., 2011). The main active components of ephedra are ephedrine and pseudoephedrine, which could exert anti-inflammatory effects by reducing the degradation of NF-κB in the cytoplasm and the production of tumor necrosis factor-α (TNF-α) (Wu et al., 2014). Among them, ephedrine has a more significant bronchial dilating effect (Laitinen et al., 1982). And pseudoephedrine, as a sympathomimetic drug, combined with emodin could regulate the polarization of macrophages to improve Lipopolysaccharide (LPS)-induced acute lung injury (Wang et al., 2022).

For the SG in MXSG, the main component is CaSO4·2H2O, also including manganese (Mn), nickel (Ni) and other trace elements. SG is mainly used in TCM to clear heat and related cough, with antipyretic and cooling effects (Lin and Gao, 1994). Studies have shown that SG has anti-inflammatory and antipyretic effects, which may be related to the reduction of hypothalamic prostaglandin E2 content, while CaSO4.2H2O has no obvious anti-inflammatory and antipyretic effects, which also explains that other trace elements may play a key role (Zhou et al., 2012). The action mechanism of SG also includes affecting the firing activity of temperature-sensitive neurons in the preoptic area of the anterior hypothalamus (PO/AH) under the action of pyrogen, and playing an antipyretic role at the level of central neurons (Fan et al., 1997; Wang et al., 2008). Animal experiments showed that the healthy and harmless rabbits with fever caused by intravenous injection of typhoid vaccine were given SG decoction orally, and the control group was given antipyrine. The results show that it does have a significant cooling effect, with a rapid cooling rate similar to antipyrine (Zhou, 2015). SG combined with MH has antipyretic and anti-asthmatic effects (Mei et al., 2016), and SG combined with Anemarrhena could exert anti-allergic effects (Makino et al., 2014). Glycyrrhizic acid, the main active ingredient in GC, has broad-spectrum antiviral activity, and glycyrrhizin could inhibit SARS-related virus replication (Cinatl et al., 2003). Glycyrrhizin could reduce viral infection of cells, mainly by reducing endocytosis of the cell membrane and reducing viral uptake (Wolkerstorfer et al., 2009). Liquiritigenin exerts anti-inflammatory effects due to inhibition of NF-κB activation in macrophages, thereby reducing Inductible Nitric Oxide Synthase and pro-inflammatory cytokine production (Kim et al., 2008). KXR is protective during epithelial-mesenchymal transition in chronic obstructive pulmonary disease mice (Wang et al., 2019). Other studies have shown that amygdalin, one of the main pharmacologically active ingredients of KXR, could inhibits NF-κB and NOD-like receptor protein 3 (NLRP3) signaling pathways, thereby reducing the expression of pro-inflammatory cytokines (such as pro-IL-1b), resulting in anti-inflammatory effects (Zhang et al., 2017).

However, our studies also have certain limitations. Could the lack of significant benefits after further adjustments in MXSG dosage be attributed to the fact that patients’ blood drug concentrations have already reached their maximum? Additionally, can the clinically established baseline dosage of MXSG be applicable to every individual patient? These questions cannot be addressed through our studies alone and may need to be answered through subsequent PK/PD experiments. Of course, MXSG is a compound composed of various plants and minerals. Although our clinical studies, including previously published research, have confirmed its significant clinical efficacy, the active ingredients and mechanism of action are yet to be determined. Furthermore, in our studies, the subjects were all Chinese. The efficacy of MXSG in other ethnic groups and the optimal effective dosage require further evaluation.

In conclusion, the results from these 3 clinical studies indicate that, for the clinical application of MXSG, the commonly used clinical dosage is optimal. Increasing the dosage of the drug may not yield significant benefits. Adjusting the dosage of SG or KXR could lead to a certain degree of improvement in fever and cough symptoms, all within the boundaries of safety. Our research provides high-quality evidence-based support for the rational clinical application and dosage selection of MXSG. Moreover, it offers insights and methods that can be referenced for determining dosages of other TCM formulas and for related research endeavors. Of course, our research is still exploratory clinical trials, and further studies are needed to confirm our findings.

## Data Availability

The original contributions presented in the study are included in the article/Supplementary Material, further inquiries can be directed to the corresponding authors.
