# Supplementary material for: The rational dose for MaXingShiGan decoction is crucial for its clinical effectiveness in treating bronchial pneumonia: three randomized, double-blind, dose-parallel controlled clinical studies
Source: Front Pharmacol. 2023 Nov 23;14:1279519. doi: 10.3389/fphar.2023.1279519 (PMC10701270; doi:10.3389/fphar.2023.1279519)
Supplement: Supplementary file 1 [file DataSheet1.docx]

**Supplementary appendix**

**1.** **The extraction process and quality control of MXSG**

**1.1** **The decoction process of MXSG in clinical studies**

Measure the herbal ingredients, add eight times the amount of water, soak for 30 minutes, bring to a boil, then reduce to low heat and simmer for 40 minutes. Let it cool, filter through gauze, and obtain the decoction.

**1.2** **Quality control of MXSG**

**(1) Selection of MXSG**

We conducted content determination for the herbal components of the purchased MXSG. This included 7 batches of Ephedra herb slices, 7 batches of Bitter Almond herb slices, 6 batches of Gypsum, and 4 batches of Licorice, in accordance with the standards set by the 《Chinese Pharmacopoeia》. Based on these standards, we determined the herbal materials to be used in the clinical study of MXSG for this project."

Table 1 Information on MXSG

| Medicinal Herbs | Place of Origin | Source | Analyzed Components | Pharmacopoeia Standards | Measured Content |
| --- | --- | --- | --- | --- | --- |
| *Ephedra sinica* Stapf | Inner Mongolia | Shuangqiao Yanjing Pharmaceutical Co., Ltd. in Beijing, China | Ephedrine | 1.0% | 1.09% |
|  |  |  | Pseudoephedrine | —— | 0.72% |
| *Prunus armeniaca* L. | North China | Shuangqiao Yanjing Pharmaceutical Co., Ltd. in Beijing, China | Prunasin | 3.0% | 3.78% |
| Gypsum Fibrosum | Hebei | Shuangqiao Yanjing Pharmaceutical Co., Ltd. in Beijing, China | Calcium sulfate dihydrate | 95% | 99.71% |
| *Glycyrrhiza glabra* L*.* | Inner Mongolia | Shuangqiao Yanjing Pharmaceutical Co., Ltd. in Beijing, China | Glycyrrhizin | 0.5% | 0.77% |
|  |  |  | Glycyrrhetinic acid | 1.0% | 2.29% |

**(2) Determination of content in MXSG**

The content determination method for MXSG mainly involves the quantitative analysis of six key components: *Ephedra sinica* Stapf (Ephedrine, Pseudoephedrine), *Prunus armeniaca* L. (Prunasin), *Glycyrrhiza glabra* L (Glycyrrhetinic acid, Glycyrrhizin), and Gypsum Fibrosum (Calcium sulfate dihydrate, Ca2^+^). Specifically, Ephedrine, Pseudoephedrine, Prunasin, Glycyrrhetinic acid, and Glycyrrhizin are quantified using High-Performance Liquid Chromatography (HPLC), while Calcium sulfate dihydrate (Ca2^+^) is determined through titration.

**Experimental Materials**

Ephedrine, Pseudoephedrine, Ammonium Glycyrrhetate, and Amygdalin were all purchased from the China National Institutes for Food and Drug Control.

**Medicinal Herbs**

Purchased from Shuangqiao Yanjing Pharmaceutical Co., Ltd. in Beijing and Jiangxi Provincial Hospital of Traditional Chinese Medicine.

**Instrumentation**

Waters Acquity Ultra-High-Performance Liquid Chromatography System (Waters Corporation, USA), Electronic Analytical Balance (AL204, Mettler-Toledo Instruments (Shanghai) Co., Ltd.), Ultrasonic Cleaner (KQ-500B, Kunshan Ultrasonic Instruments Co., Ltd.).

**Detection Method**

Preparation of Reference Solution: Dissolve hydrochloride ephedrine reference substance and hydrochloride pseudoephedrine reference substance in methanol to obtain solutions with concentrations of 20μg/ml each. Dissolve glycyrrhizin reference substance and ammonium glycyrrhetate reference substance in 70% ethanol to obtain solutions with concentrations of 20μg/ml and 0.2mg/ml, respectively. Dissolve amygdalin reference substance in methanol to obtain a solution with a concentration of 40μg/ml.

Preparation of MXSG: Take 20g of MH, 15g of KXR, 40g of SG, and 10g of GC. Add 680ml of water, soak for 30 minutes, heat to a boil over high heat, then reduce to a low simmer. Filter the mixture.

Preparation of MXSG Test Solution: Take 10ml of the decoction, place it in a 100ml volumetric flask, add an appropriate amount of methanol, perform ultrasonic treatment for 15 minutes, allow it to stand for 1 hour, dilute with methanol to volume, shake well, and filter through a 0.45μm microporous membrane.

Chromatographic Conditions: Agilent 1100-DAD detector, Diamonsil C18 column (250mm*4.6mm, 5μm). The mobile phase consists of acetonitrile and 0.1% phosphoric acid with a gradient elution. The detection wavelengths are as follows: 0 to 32 minutes at 215nm, 32 to 40 minutes at 350nm, and 40 to 50 minutes at 254nm. The injection volume is 10μl, column temperature is set at 25°C, and the flow rate is 1ml/min. A gradient elution program is used."

Table 2 The Gradient Elution Program for MXSG Mobile Phase

| Time (min) | Acetonitrile (%) | 0.1 Phosphoric acid-water (%) |
| --- | --- | --- |
| 0 | 4 | 96 |
| 12 | 5 | 95 |
| 18 | 17 | 83 |
| 23 | 19 | 81 |
| 50 | 70 | 30 |
| 60 | 100 | 0 |

"Through the analysis of the HPLC fingerprint profiles of 10 batches of MXSG test solutions, a total of 12 common peaks were identified. In the fingerprint profiles, peak number 8 corresponded to glycyrrhizin, which was used as a reference peak. The relative retention times of the common peaks in the 10 batches of samples were calculated, and the results showed that the retention time range was less than 3%, meeting the requirements of the fingerprint profiles.

Using Chinese herbal fingerprint similarity calculation software, the similarity results indicated that the fingerprint profiles of the 10 batches of MXSG were all greater than 0.95, demonstrating the stability of the standard decoction preparation process (Figure 1).


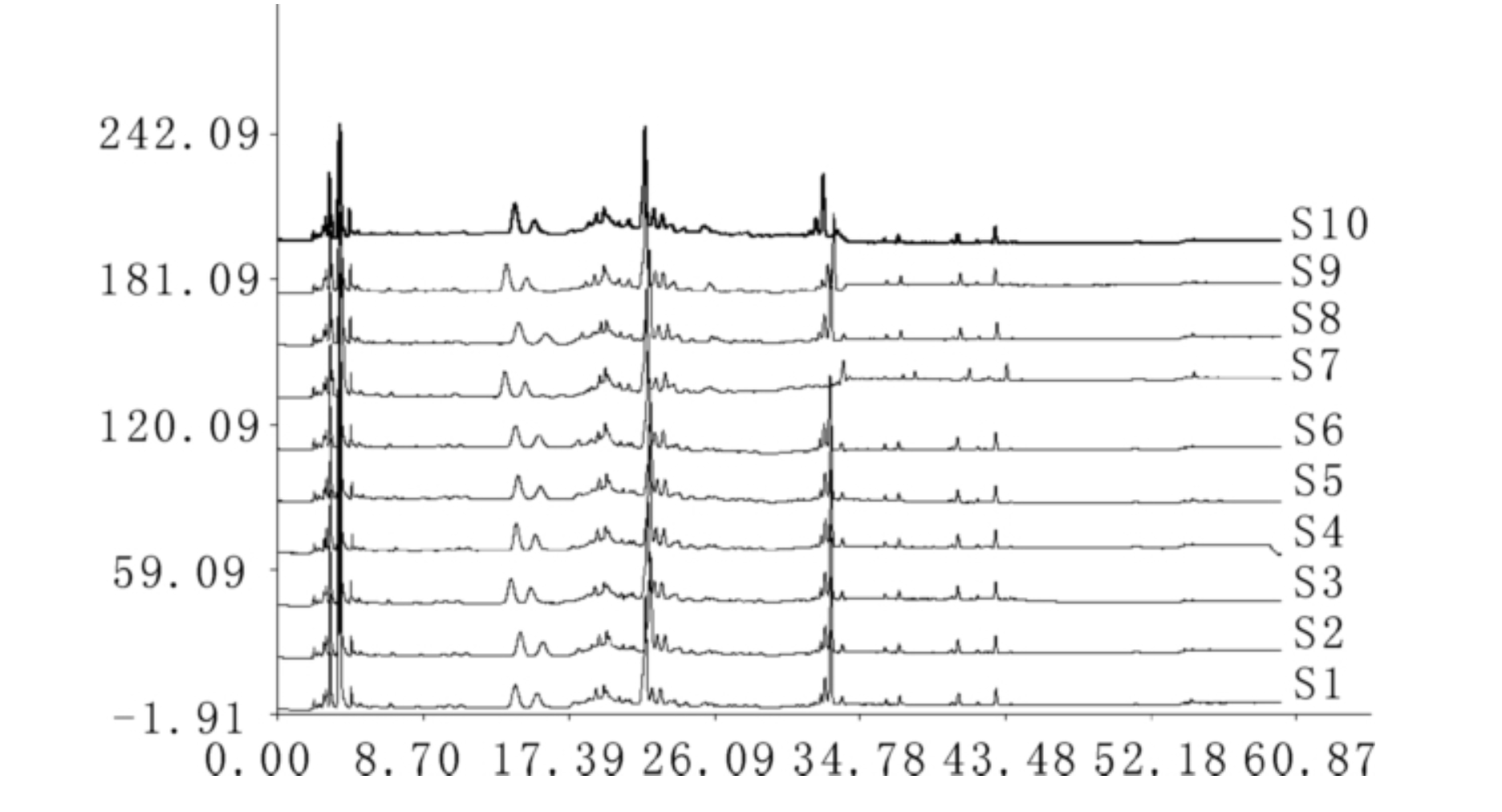


Figure 1 Multi-Wavelength Switching Fingerprint Profiles of 10 Batches of MXSG

We identified specific fingerprint peaks in the test solution of MXSG. Among them, peak 1 was ephedrine, peak 2 was pseudoephedrine, peak 4 was prunasin, peak 8 was glycyrrhizin, and peak 12 was glycyrrhetinic acid. (Figure 2)


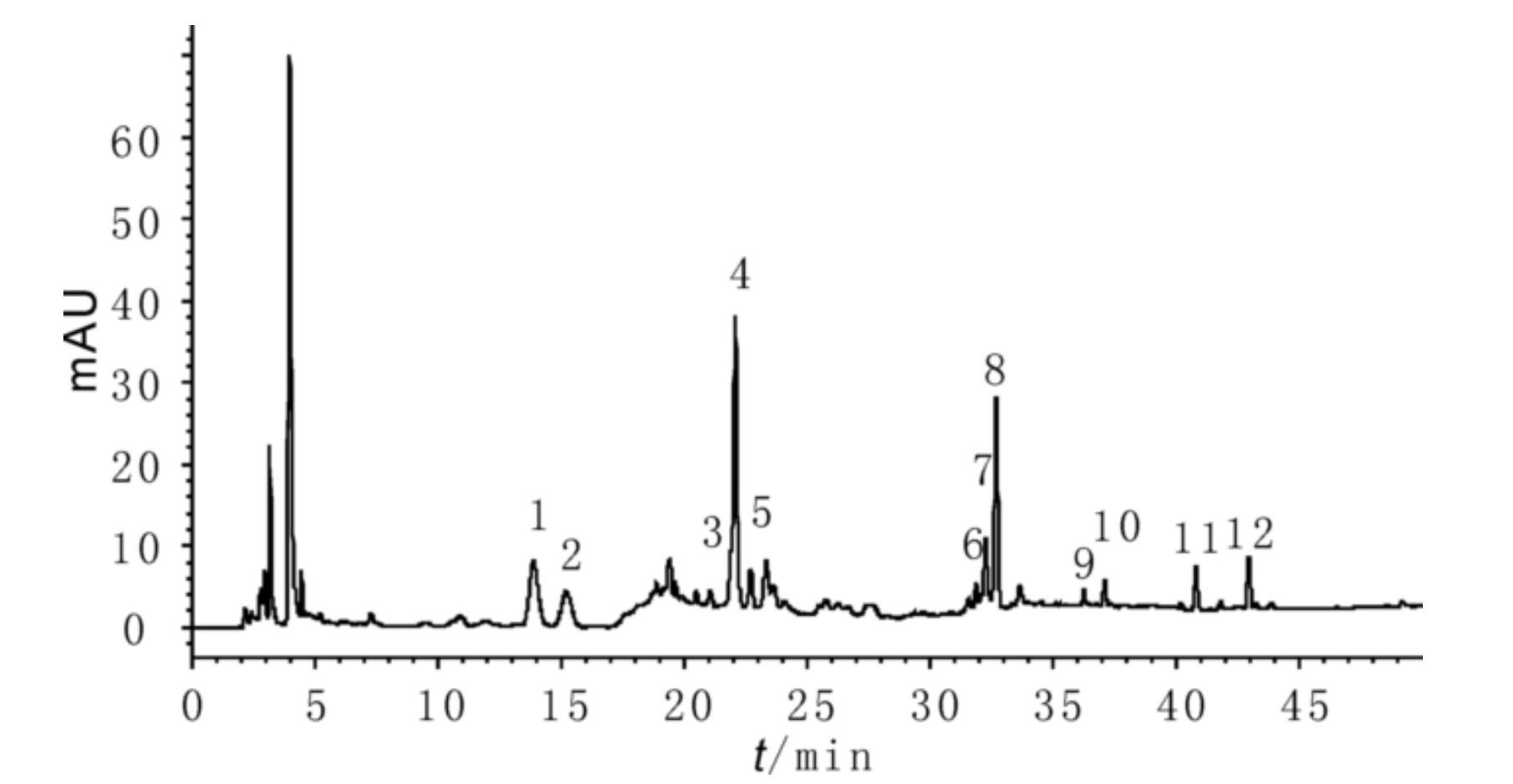


Figure 2 Identification of Some Fingerprint Peaks in MXSG

**(3) Determination of blood absorption components in MXSG**

We administered the drugs to mice in 10 separate groups (n=3 each) via oral gavage and measured the levels of active components in the mice's blood samples.

Mass Spectrometry Conditions: LC-MS-MS (Finnigan, USA)

Analytes: This included ephedrine, pseudoephedrine, methyl ephedrine, prunasin, glycyrrhizin, glycyrrhetinic acid, glycyrrhetinic acid, glycyrrhetin, and isoglycyrrhetin.

Through secondary and tertiary feature peak comparisons, we were able to confirm the presence of ephedrine or pseudoephedrine, methyl ephedrine, glycyrrhizin, glycyrrhetin, and isoglycyrrhetin in the blood samples. We primarily compared pseudoephedrine, methyl ephedrine, and glycyrrhizin. Glycyrrhetinic acid, glycyrrhetinic acid, and prunasin were not detected. (Figures 3-8)

Figure 3 Sample secondary spectrum (1)

Figure 4 Pseudoephedrine secondary spectrum

Figure 5 Sample secondary spectrum (2)

Figure 6 Methyl Ephedrine secondary spectrum

Figure 7 Sample secondary spectrum (3)

Figure 8 Glycyrrhizin secondary spectrum

**2.** **Toxicity experiment of MXSG**

"We selected 50 healthy C57 mice with a weight range of 18 to 22g and randomly divided them into 9 groups: a blank control group, a group receiving a dose of 240g of raw MXSG per kg of body weight, a group receiving a dose of 168g/kg, a group receiving a dose of 116g/kg, a group receiving a dose of 80g/kg, a group receiving a dose of 56g/kg (equivalent to 87.5 times the human daily dose), a group receiving a dose of 39.2g/kg (equivalent to 64.3 times the human daily dose), a group receiving a dose of 27.4g/kg (equivalent to 42.8 times the human daily dose), and a group receiving a dose of 19.2g/kg (equivalent to 30 times the human daily dose). Each group consisted of 10 mice, evenly split between males and females.

On the day of administration and particularly within 4 hours after dosing, the mice were closely observed. Subsequently, observations were made twice daily (morning and afternoon) for 14 consecutive days. This included monitoring changes in animal body weight, diet, behavior, secretions, excretions, symptoms of abnormal reactions, onset time, severity, duration, reversibility, and mortality.

No deaths were observed in mice given doses below 56g/kg (equivalent to 87.5 times the human daily dose). The study results indicated that there were no apparent toxic reactions in mice after a single oral administration of MXSG. The observed LD50 value after a 15-day observation period was 160.64g/kg of raw material (with a 95% confidence interval of 128.39 to 222.72g/kg)."

**3. The flowchart of the clinical study process**


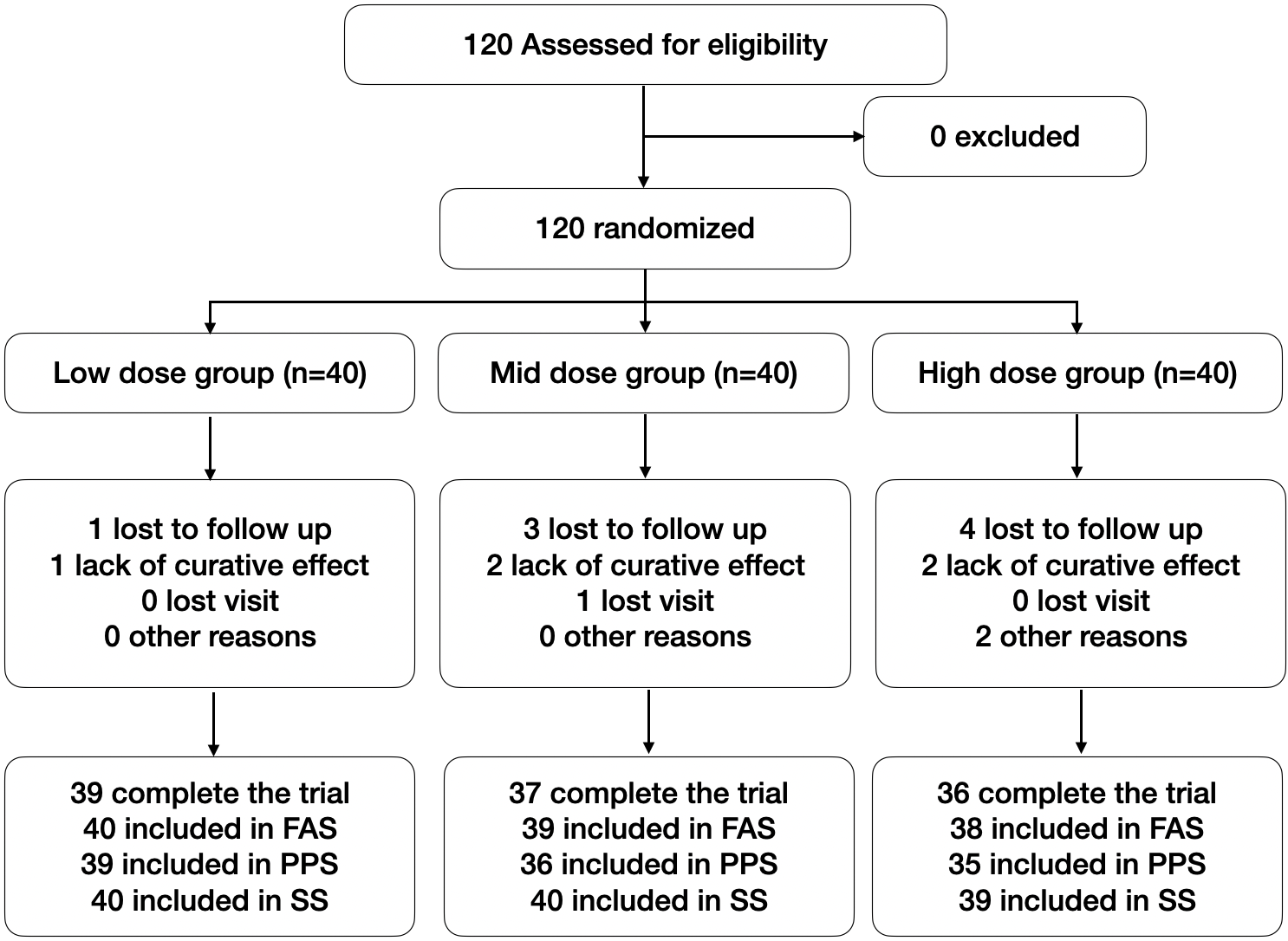


Figure 1 Flowchart of Study 1


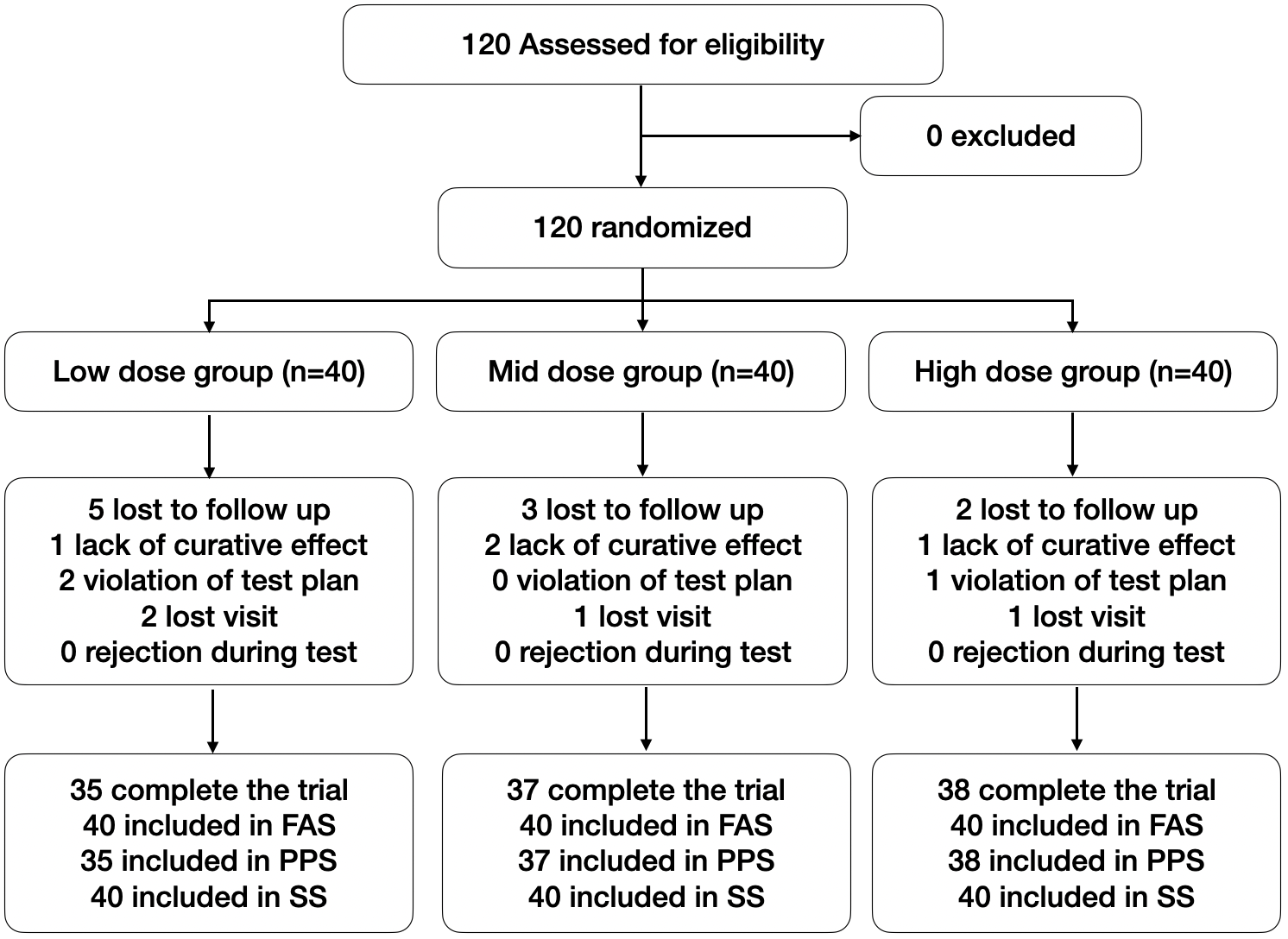


Figure 2 Flowchart of Study 2


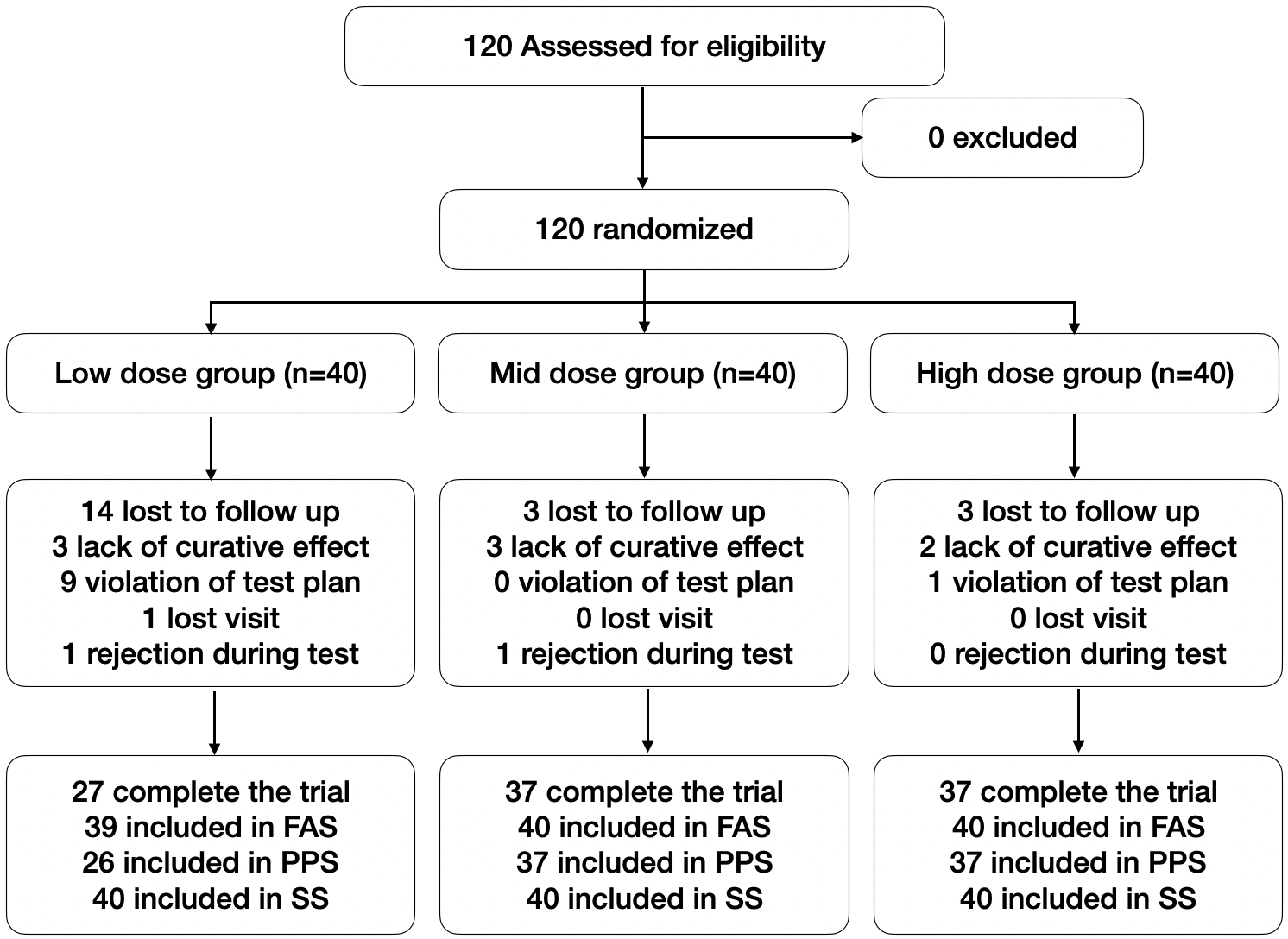


Figure 3 Flowchart of Study 3

**4.The dose of MXSG in different studies**

Table 1 The dose of MXSG in different groups of study 1 (g)

| Name | Chinese name | Part used | Low dose group | mid dose group | High dose group |
| --- | --- | --- | --- | --- | --- |
| *Ephedra sinica* Stapf | MaHaung | Dried stems and leaves | 3 | 6 | 9 |
| *Prunus armeniaca* L. | KuXingRen | Dried seeds | 3 | 6 | 9 |
| Gypsum fibrosum | ShiGao | CaSO4 • 2H2O | 12 | 24 | 36 |
| *Glycyrrhiza uralensis* Fisch. | GanCao | Dried roots and leaves | 3 | 6 | 9 |

Table 2 The dose of MXSG in different groups of study 2 (g)

| Name | Chinese name | Part used | Low dose group | | mid dose group | High dose group |
| --- | --- | --- | --- | --- | --- | --- |
| *Ephedra sinica* Stapf | MaHaung | Dried stems and leaves | 6 | 6 | | 6 |
| *Prunus armeniaca* L. | KuXingRen | Dried seeds | 6 | 6 | | 6 |
| Gypsum fibrosum | ShiGao | CaSO4 • 2H2O | 16 | 24 | | 36 |
| *Glycyrrhiza uralensis* Fisch. | GanCao | Dried roots and leaves | 6 | 6 | | 6 |

Table 3 The dose of MXSG in different groups of study 3 (g)

| Name | Chinese name | Part used | Low dose group | mid dose group | High dose group |
| --- | --- | --- | --- | --- | --- |
| *Ephedra sinica* Stapf | MaHaung | Dried stems and leaves | 6 | 6 | 6 |
| *Prunus armeniaca* L. | KuXingRen | Dried seeds | 36 | 6 | 9 |
| Gypsum fibrosum | ShiGao | CaSO4 • 2H2O | 24 | 24 | 24 |
| *Glycyrrhiza uralensis* Fisch. | GanCao | Dried roots and leaves | 6 | 6 | 6 |

**5. Diagnostic Criteria for Pediatric Bronchopneumonia**

Based on the 'Prevention and Treatment Plan for Pediatric Four Diseases·Prevention and Treatment Plan for Pediatric Pneumonia' by the Ministry of Health of the People's Republic of China, formulated according to the 7th edition of 'Practical Pediatric Science' by Zhu Futang.

(1) General Symptoms: Onset can be sudden or gradual, with abrupt symptoms such as fever, refusal to eat, vomiting, excessive sleepiness, irritability, wheezing, and other symptoms.

(2) Respiratory System Symptoms and Signs: Symptoms include coughing with phlegm sound in the throat, increased breathing rate, often accompanied by difficulty in breathing. Severe cases may show groaning sounds during exhalation, flaring of the nostrils, intercostal retractions, perioral cyanosis, or cyanosis of the nailbeds. Chest signs may not be obvious early on, but later, medium-to-coarse wet rales may be heard, followed by fine wet rales or crepitating sounds after a few days. Tubular breathing sounds might also be detected.

(3) Chest X-ray: Can reveal non-specific patchy infiltrates in lung parenchyma, often more prominent in the lower lungs, cardiac-diaphragmatic angle region, and mid-zones. These infiltrates may partially merge into larger patches.

(4) Blood Test: White blood cell count may decrease, remain normal, or slightly increase."

**6.** **Traditional Chinese Medicine Syndrome Diagnostic Criteria for Wind-Heat Constrained in the Lungs in Pediatric Bronchopneumonia**

Formulated according to the National Administration of Traditional Chinese Medicine's 'Diagnostic and Therapeutic Criteria of TCM Patterns and Diseases·Diagnostic and Therapeutic Criteria of Pediatric TCM Patterns and Diseases' for the categorization of the wind-heat constrained in the lungs pattern in pediatric pneumonia and cough.

Wind-Heat Constrained in the Lungs Pattern: Fever with aversion to wind, slight perspiration, thirst with a desire to drink, cough, thick yellow phlegm, rapid breathing, and redness in the throat. The tip of the tongue is red, the coating is thin and yellow, and the pulse is floating and rapid."

**7. Clinical syndrome scoring and grading quantitative standard**

Table 4 Clinical syndrome scoring and grading quantitative standard

| **Symptoms** | **0 point (asymptomatic)** | **2 points**  **(mild)** | **4 points**  **(moderate)** | **6 points**  **(severe)** |
| --- | --- | --- | --- | --- |
| **Fever** | <37.3℃ | 37.3～38℃ | 38.1～38.5℃ | >38.5℃ |
| **Cough** | none | Intermittent cough during the day, mild | Cough frequently, but does not affect sleep | Cough frequently or intermittently day and night, affecting rest and sleep |
| **Dyspnea** | Breathing is stable and the number of times is within the normal range of corresponding age | Exceeding the upper limit of the normal value of the corresponding age ≤ 10 times / minute, without nasal fan and three concave signs | Exceed the upper limit of the normal value of the corresponding age by 11 ~ 20 times / minute, and / or intermittent wheezing, nasal fan and three concave signs | Exceeding the upper limit of normal value of corresponding age ≥ 21 times / minute, and / or continuous wheezing, nasal fan and three concave signs |
| **Phlegm Obstruction** | None | Less phlegm, occasional phlegm and stridor in the throat | There is phlegm, sometimes there is phlegm in the throat | Phlegm is much, yellow or sticky, and the phlegm sound in the throat is wet |
| **Lungs**  **sign** | Clear breath sound | The breathing sound is reduced, or rough, or medium wet rale is heard | Moderate and fine moist rales in one lung, and / or little wheezing | Moderate and fine moist rales in both lungs, and / or significant wheezing |

**8. Drug Safety Assessment**

(1) Incidence of Adverse Reactions: Record adverse reactions (symptoms, signs, laboratory tests, etc.) that occur during the treatment process with different treatment regimens. Evaluate the timing, duration, severity, measures taken, course of events, and assess their correlation with the medication.

(2) Safety Assessment: Statistically compare the occurrence of adverse reactions with different treatment regimens in this study and provide safety assessments for each treatment regimen.

(3) Criteria for Judging Severity of Adverse Events

Mild: Subjects can tolerate, does not affect treatment, does not require special intervention, and has no impact on subject recovery.

Moderate: Subjects find it difficult to tolerate, requires discontinuation of the investigational drug or special intervention, and has a direct impact on subject recovery.

Severe: Endangers the subject's life, leads to death or disability, requires immediate discontinuation of the drug or emergency intervention.

**9. Baseline Characteristics of Different Studies**

**Table 5 Baseline Characteristics of study 1**

| **Contents** | **Low dose group (n=40)** | **Mid dose group (n=39)** | **High dose group (n=38)** | **Test statistics** | ***P* value** |
| --- | --- | --- | --- | --- | --- |
| Age (years) | 4.31±0.92 | 4.22±0.87 | 4.43±1.01 | 0.51 (F value) | 0.6040 |
| Male | 20 (50.00%) | 21 (53.85%) | 15 (39.47%) | 1.70 (Chi square) | 0.4265 |
| Female | 20 (50.00%) | 18 (46.15%) | 23 (60.53%) |  |  |
| Height (cm) | 105.38±8.24 | 105.56±7.62 | 105.71±7.66 | 0.02 (F value) | 0.9828 |
| Weight (kg) | 18.45±3.57 | 18.26±2.64 | 18.39±3.99 | 0.03 (F value) | 0.9676 |
| Disease Duration（h） | 10.68±11.12 | 11.64±12.05 | 8.29±10.15 | 3.27 (Kruskal-Wallis) | 0.1953 |
| Temperature (℃) | 37.50±0.91 | 37.50±0.82 | 37.47±0.88 | 0.01 (F value) | 0.9883 |
| Heart Rate | 111.25±10.73 | 106.00±10.65 | 112.08±14.78 | 2.85 (F value) | 0.0619 |
| Respiratory Rate | 26.93±4.25 | 26.23±3.84 | 27.47±3.98 | 0.92 (F value) | 0.4015 |
| **Blood Pressure** |  |  |  |  |  |
| SBP (mmHg) | 85.13±3.84 | 84.62±4.20 | 85.79±3.59 | 0.88 (F value) | 0.4161 |
| DBP (mmHg) | 53.13±6.76 | 52.46±6.88 | 53.89±5.71 | 0.47 (F value) | 0.6257 |
| **Comorbid Symptoms** |  |  |  |  |  |
| Fever (n) | 4.80±1.56 | 4.26±1.67 | 4.37±1.73 | 2.40 (F value) | 0.3007 |
| Cough (n) | 4.05±1.06 | 4.10±1.12 | 4.26±1.33 | 0.79 (F value) | 0.6737 |
| Dyspnea | 1.00±1.11 | 0.82±1.10 | 1.32±1.16 | 3.86 (F value) | 0.1454 |
| Phlegm Obstruction | 3.60±0.81 | 3.33±1.24 | 3.63±1.02 | 1.83(F value) | 0.4003 |
| Lung Signs | 3.60±1.65 | 3.59±1.53 | 3.74±1.55 | 0.25 (F value) | 0.8840 |

**Table 6 Baseline Characteristics of study 2**

| **Contents** | **Low dose group (n=40)** | **Mid dose group (n=40)** | **High dose group (n=40)** | **Test statistics** | ***P* value** |
| --- | --- | --- | --- | --- | --- |
| Age (years) | 4.38±1.07 | 4.32±1.01 | 4.42±1.08 | 0.09 (F value) | 0.9100 |
| Male | 19(47.50%) | 23(57.50%) | 31(77.50%) | 7.83 (Chi square) | 0.0199 |
| Female | 21(52.50%) | 17(42.50%) | 9(22.50%) |  |  |
| Height (cm) | 104.38±9.18 | 103.90±9.79 | 104.48±10.03 | 0.04 (F value) | 0.9605 |
| Weight (kg) | 18.10±3.35 | 18.20±3.61 | 17.80±3.28 | 0.15 (F value) | 0.8622 |
| Disease Duration（h） | 25.13±16.29 | 26.65±15.80 | 23.28±15.75 | 0.43 (Kruskal-Wallis) | 0.8074 |
| Temperature (℃) | 37.37±0.70 | 37.70±0.75 | 37.62±0.82 | 2.11 (F value) | 0.1259 |
| Heart Rate | 105.35±12.15 | 106.40±12.02 | 105.05±10.08 | 0.15 (F value) | 0.8581 |
| Respiratory Rate | 25.73±2.86 | 26.38±2.68 | 25.53±3.13 | 0.94 (F value) | 0.3921 |
| **Blood Pressure** |  |  |  |  |  |
| SBP (mmHg) | 90.88±8.46 | 90.53±8.24 | 93.00±7.49 | 1.10 (F value) | 0.3364 |
| DBP (mmHg) | 59.25±6.26 | 58.50±6.52 | 60.75±5.61 | 1.39 (F value) | 0.2528 |
| **Comorbid Symptoms** |  |  |  |  |  |
| Fever (n) | 5.15±1.00 | 5.50±0.88 | 5.45±0.90 | 1.66 (F value) | 0.1945 |
| Cough (n) | 4.25±1.03 | 4.30±1.07 | 4.50±1.18 | 0.59 (F value) | 0.5585 |
| Dyspnea | 1.15±1.19 | 1.10±1.19 | 0.95±1.11 | 0.32 (F value) | 0.7270 |
| Phlegm Obstruction | 3.40±1.03 | 3.75±1.03 | 3.60±0.93 | 1.24 (F value) | 0.2942 |
| Lung Signs | 3.90±1.35 | 4.05±1.40 | 3.70±1.40 | 0.64 (F value) | 0.5268 |

**Table 7 Baseline Characteristics of study 3**

| **content** | **low dose group (n=39)** | **mid dose group (n=40)** | **high dose group (n=40)** | **Test statistics** | ***P* value** |
| --- | --- | --- | --- | --- | --- |
| Age (years) | 4.09±0.85 | 4.46±0.97 | 4.06±0.85 | 2.39 (F value) | 0.0959 |
| Male | 27(69.23%) | 20(50.00%) | 20(50.00%) | 3.94 (Chi square) | 0.1394 |
| Female | 12(30.77%) | 20(50.00%) | 20(50.00%) |  |  |
| Height (cm) | 103.90±7.62 | 106.60±8.50 | 104.43±6.82 | 1.38 (F value) | 0.2551 |
| Weight (kg) | 18.09±4.53 | 18.85±4.03 | 17.62±3.04 | 1.01 (F value) | 0.3666 |
| Disease Duration（h） | 20.05±14.49 | 23.25±14.41 | 21.18±13.22 | 0.96 (Kruskal-Wallis) | 0.6186 |
| Temperature (℃) | 37.21±0.87 | 37.08±0.68 | 37.44±0.94 | 1.83 (F value) | 0.1644 |
| Heart Rate | 100.51±10.60 | 100.08±9.08 | 101.43±7.22 | 0.23 (F value) | 0.7939 |
| Respiratory Rate | 24.64±2.27 | 24.55±2.16 | 24.88±3.05 | 0.18 (F value) | 0.8386 |
| **Blood Pressure** |  |  |  |  |  |
| SBP (mmHg) | 92.82±7.05 | 93.38±7.49 | 91.30±6.58 | 0.93 (F value) | 0.3982 |
| DBP (mmHg) | 60.90±6.48 | 60.33±5.18 | 59.13±3.90 | 1.16 (F value) | 0.3161 |
| **Comorbid Symptoms** |  |  |  |  |  |
| Fever (n) | 1.44±2.29 | 1.50±1.80 | 2.15±2.33 | 1.34 (F value) | 0.2653 |
| Cough (n) | 4.88±1.00 | 4.80±0.99 | 4.75±0.98 | 0.15 (F value) | 0.8608 |
| Dyspnea | 0.05±0.32 | 0.25±0.81 | 0.15±0.53 | 1.12 (F value) | 0.3308 |
| Phlegm Obstruction | 4.00±0.65 | 3.80±0.88 | 3.80±0.76 | 0.88 (F value) | 0.4158 |
| Lung Signs | 3.38±1.23 | 3.40±1.22 | 3.60±1.37 | 0.35 (F value) | 0.7028 |

**10. Summary of Adverse Event Results in Each Study**

| Study | Group | adverse event | Duration | Severity | Outcome | Taking measures | Corrective treatment | Whether withdraw | Relationship with the investigational drug | Adverse reactions of investigational drug |
| --- | --- | --- | --- | --- | --- | --- | --- | --- | --- | --- |
| Study 1 | Mid dose group | Vomiting, abdominal pain | 1 day | Mild | Disappeared | Completely discontinued | None | Yes | Impossible | No |
|  |  | Palpitations, restlessness | 5 days | Mild | Disappeared | Continued use | None | No | Possible | Yes |
|  | High dose group | Otitis media | 3 days | Mild | Relieved | Continued use | Ofloxacin eye drops for ear instillation | No | Impossible | No |
|  |  | Allergic reaction | 3 days | Mild | Disappeared | Continued use | Montelukast, 5mg per dose, once daily | No | Impossible | No |
|  |  | Skin rash | 3 days | Mild | Disappeared | Continued use | Montelukast, 5mg per dose, once at night | No | Impossible | No |
| Study 2 | High dose group | Skin rash | 3 days | Mild | Disappeared | Continued use | Promethazine, Dexamethasone | No | Impossible | No |

**Table 8 Summary of Adverse Event Results in Each Study**
